# Alleged Poisoning by the Vapour of Phosphorus

**Published:** 1846-12

**Authors:** 


*Alleged Poisoning by the Vapour of Phosphorus.*—According to M. Dupasquier, the effects of the vapour of phosphorus among workmen employed in the manufacture of lucifer matches have been much exaggerated. He has remarked that these persons were subject only to slight attacks of bronchitis. The more serious symptoms recorded by some observers, were, he considers, due to the improper admixture of arsenic with the phosphorus paste.—*Ibid.*

